# Quantitative pedigree analysis and mitochondrial DNA sequence variants in adults with cyclic vomiting syndrome

**DOI:** 10.1186/1471-230X-14-181

**Published:** 2014-10-21

**Authors:** Thangam Venkatesan, Essam A Zaki, Nilay Kumar, Jyotirmoy Sengupta, Muhammad Ali, Baber Malik, Aniko Szabo, Miranda AL van Tilburg, Richard G Boles

**Affiliations:** Division of Gastroenterology and Hepatology, Medical College of Wisconsin, Milwaukee, WI USA; Division of Medical Genetics, Children’s Hospital Los Angeles, Los Angeles, CA USA; Division of Gastroenterology and Hepatology, UT Southwestern Medical Center, Dallas, TX USA; Division of Biostatistics, Institute for Health and Society, Medical College of Wisconsin, Milwaukee, WI USA; Center for Functional GI and Motility Disorders, The University of North Carolina at Chapel Hill, Chapel Hill, NC USA; Department of Pediatrics, Keck School of Medicine at the University of Southern California, Los Angeles, CA USA

**Keywords:** Vomiting, Mitochondria, Pedigree, Genetic, Functional disorders

## Abstract

**Background:**

Children with cyclic vomiting syndrome (CVS) have a high degree of maternal inheritance of functional gastrointestinal and neurological disorders. CVS in children is also associated with an increased prevalence of mitochondrial DNA single-nucleotide polymorphisms (mtDNA SNPs) 16519 T and 3010A. Preliminary data suggests that age of onset of symptoms (pediatric vs. adult) may be a determinant of the presence of such mtDNA SNP’s. We sought to examine the degree of maternal inheritance pattern of functional disorders and the prevalence of mtDNA SNP’s16519T and 3010A in adults with CVS and correlate this with age of onset of disease.

**Methods:**

A Quantitative Pedigree Analysis (QPA) was performed in 195 of a total of 216 patients and all were genotyped using Restriction Fragment Length Polymorphism (RFLP) or sequencing.

**Results:**

Adults with CVS had a higher degree of probable maternal inheritance (PMI) of functional disorders than controls (12% vs. 1%, p < 0.001). However, the prevalence of mitochondrial SNP’s 16519 T, 3010A and the AT genotype were similar in Haplogroup H CVS patients compared to historical controls. There was no correlation between age of onset of disease and prevalence of these mtDNA SNP’s.

**Conclusions:**

A subset of adults with CVS has a significantly higher degree of maternal inheritance pattern of functional disorders than controls. There was no association with mtDNA SNP’s 16519 T and 3010A as seen in children and future studies sequencing the entire mitochondrial and nuclear genome to identify potential causes for this maternal inheritance pattern in adults are warranted.

**Electronic supplementary material:**

The online version of this article (doi:10.1186/1471-230X-14-181) contains supplementary material, which is available to authorized users.

## Background

Cyclic vomiting syndrome (CVS) is a chronic disorder characterized by episodic nausea and vomiting and is diagnosed using Rome criteria [1]. Subsets of CVS include CVS plus and catamenial CVS. CVS plus is defined by the presence of at least two neuromuscular disorders in association with CVS [2]. CVS can sometimes occur in association with the menstrual cycle and this is referred to as catamenial CVS [3]. The pathophysiology of CVS is not known but preliminary reports in children suggest that genetic factors play an important role. Extensive pedigree analysis of children with CVS has revealed a clustering of functional disorders in matrilineal relatives [4, 5]. There is a lack of similar data on matrilineal inheritance patterns in adults with CVS. Further, analysis of the mitochondrial genome in children has shown an increased frequency of two mitochondrial DNA polymorphisms 16519 T and 3010A, which together confer 17-fold higher odds of having CVS in comparison to controls [6]. The 16519 T polymorphism alone was associated with 6-fold higher odds of CVS [6]. Experts consider CVS as part of a migraine diathesis and mitochondrial single-nucleotide polymorphisms (SNP’s) have been associated with migraines as well [7].

Preliminary data by Boles et al. also suggests that CVS is genetically distinct in those with onset of symptoms < 12 years (pediatric-onset) and those > 18 years (adult–onset). A previous study showed that the association between the 16519 T and 3010A polymorphisms (the AT genotype) reported in children was absent in adults, and it was speculated that the pathophysiology of CVS may differ based on the age of onset of symptoms. This same study also found that 56% of adults with CVS had high Karolinska Scales of Personality (KSP) scores which are both sensitive and specific for mitochondrial dysfunction; findings not reconciled by the same study [8]. And so while there is some thought that CVS is genetically distinct based on onset of disease, the association with migraine, high KSP scores in adult-onset CVS which are specific and sensitive for mitochondrial dysfunction and similar response to treatment suggests otherwise and underscores the need for further analysis [9, 10].

CVS remains a clinical challenge given the absence of a biochemical marker and indeed the validity of this diagnosis is questioned by some. Thus it is imperative that we find surrogate markers for this disease; the presence of a biological marker would enable physicians to diagnose patients with a simple blood test and avoid expensive investigations to exclude various other disorders. This study is also relevant in the treatment of this disorder. CVS is currently treated with tricyclic antidepressants and mitochondrial supplements such as coenzyme Q 10 and L-carnitine. Small trials and anecdotal data showed that high doses of co-enzyme Q10, riboflavin and L-carnitine have potential efficacy in CVS [11]. Identification of mitochondrial heteroplasmies may allow us to develop therapies towards mitochondrial dysfunction to treat this and other related disorders.

We thus sought to perform a Quantitative Pedigree Analysis (QPA) using a standard questionnaire and estimate the matrilineal inheritance pattern of various functional disorders in adults with CVS. We also sought to identify the frequency of mtDNA SNP’s, 16519 T and 3010A in adults and determine if there are any significant associations with inheritance patterns and age of onset of disease.

## Methods

Patients who met Rome III criteria for CVS were recruited through the CVS Clinic at the Medical College of Wisconsin. Other potential subjects were also recruited through the Cyclic Vomiting Syndrome Association (CVSA) website and message board. All subjects not seen at the Medical College of Wisconsin (MCW) were invited to contact the principal investigator’s office at MCW in person, by e-mail or postal mail in order to participate, and only those who met Rome III criteria for CVS were included. Extensive clinical data was collected using the standard questionnaire employed by the CVS clinic at MCW and saliva samples were collected in person or mailed in by the subject. The Institutional Review Boards at the Medical College of Wisconsin, University of North Carolina (UNC) and the Children’s Hospital of LA (CHLA) all approved the study. Informed consent was obtained from all subjects and a legal guardian when applicable. All subjects were > 18 years of age at the time of the study.

Saliva was collected directly by the CVS probands using a commercially available kit (Oragene, DNA Genotek Inc., Ottawa, Ontario) and then mailed to Children’s Hospital Los Angeles (CHLA) where genetic analysis was performed. The presence or absence of the 7028C polymorphism that defines haplogroup H was determined in subjects either by cyclosequencing or by RFLP following AluI digestion, as per our routine practice. Both methods were used but sequencing was used during the latter part of the study as this was more readily available and less expensive than RFLP. These methods were extensively cross-validated in RB’s laboratory. All SNP’s, including all sequence variation in the CVS subjects and any SNP’s identified were correlated with the extensive clinical data obtained.

A detailed pedigree analysis was performed using a standard scripted questionnaire (see Additional file 1). The number of functional conditions per matrilineal relative and patrilineal relative were calculated and the maternal ratio and maternal inheritance ratio were calculated using a previously published tool called Quantitative Pedigree Analysis (QPA) which is used to assesses patterns of inheritance [12]. A patient had a probable maternal inheritance (PMI) pattern if the maternal ratio was ≥1.75 and the maternal inheritance ratio (MIR) is ≥ 3 where the MIR is calculated by dividing the maternal and paternal ratios. The inheritance pattern was defined as being indeterminate (Ind) if the maternal ratio was 1.5 - <1.75 and/or the MIR was 2 - <3, and as probable non-maternal inheritance (PNMI) if either the maternal ratio was <1.5 and/or the MIR is < 2. If the patient had cognitive impairment one or both parents/caregivers were asked to provide the information on the patient’s behalf.

Quantitative Pedigree Analysis was performed in 195/216 patients with cyclic vomiting syndrome who were genotyped and 102 healthy controls. Healthy controls were recruited through mass electronic mail among the faculty, staff and students at the University of North Carolina (UNC), as well as through the UNC Center for Functional GI and Motility Disorder registry of study participants, and the International Foundation of Functional Gastrointestinal Disorders website.

Our study group was characterized based on the onset of symptoms; pediatric-onset CVS was defined by onset of stereotypical vomiting episodes at <12 years of age, adolescent-onset CVS if episodes began between 12 and 18 years of age, and adult-onset CVS if episodes began ≥18 years of age. Adolescent onset CVS patient were excluded from the analysis as they likely represent an indeterminate group given the wide variation in physical, emotional and cognitive maturity during this period.

### Statistical analysis

Continuous variables were summarized using median and inter-quartile ranges, and between-group comparison was done using the Wilcoxon-Mann–Whitney test for two groups, and Kruskal-Wallis test for three or more groups. Categorical variables were summarized using proportions, and comparisons were done using Fisher’s exact test for binary and nominal outcomes, and proportional odds likelihood ratio test for ordered outcomes. A p-value of less than 0.05 was considered significant for our analysis. The prevalence of a genotype between CVS subgroups and controls was compared and odds ratios with 95% CI was reported. The type I error rate was controlled at 5% using a single step multiple testing adjustment based on the multivariate normal distribution in the context of a logistic regression model. Data was analyzed using R 2.13.1.

## Results

### Study subjects

Our study population consisted of 216 adults with CVS; 16% (35) had pediatric-onset CVS, 14% (30) had adolescent-onset CVS and 70% (151) had adult-onset CVS. Table 1 depicts the demographics and baseline characteristics of patients with CVS based on age of onset. The majority of patients from all three groups were Caucasian and from the United States. The median age of patients in these 3 groups were 26, 22 and 39 years, respectively; adult-onset CVS patients were likely to be older at the time of study (p value <0.001). Adult-onset patients were also less likely to be female (62% vs. 86% and 80%, p = 0.008) and have a lower incidence of neurocognitive defects (CVS+) (0% vs. 12% and 7%, p = 0.001) when compared to pediatric onset CVS. Most patients had a personal and family history of migraine and the three groups did not differ with regards to severity of disease, disability, use of any prophylactic medication for CVS, or response to standard therapy. A significant proportion of patients (28%) had undergone unnecessary surgery for CVS symptoms.

**Table 1:** **Baseline characteristics of CVS patients based on time of onset of symptoms**

Variables	Available N	Pediatric N-35	Adolescent N-30	Adults N-151	P-value
Age (Years)	216	26	22	39	<0.001^┼^**
Gender	216				0.006^¥^*
Male		14% (5)	20% (6)	38% (58)	
Female		86% (30)	80% (24)	62% (93)	
Race	196				0.293^¥^
White		100%	89% (25)	96% (129)	
Black		(33)	0% (0)	0% (0)	
Hispanic		0% (0)	7% (2)	3% (4)	
Other		0% (0)	4% (1)	1% (2)	
Hispanic Other					
Geographical Location	189				0.024^¥^*
Canada		4% (1)	0% (0)	0% (0)	
Midwest		56% (15)	76% (22)	74% (98)	
Northeast		22% (6)	3% (1)	4% (5)	
South		7% (2)	7% (2)	13% (17)	
United Kingdom		4% (1)	0% (0)	1% (1)	
West		7% (2)	14% (4)	9% (12)	
Type of CVS	181				0.001^¥^*
CVS Plus		12% (3)	7% (2)	0% (0)	
Classical CVS		76% (19)	76% (22)	93% (118)	
Catamenial CVS		12% (3)	17% (5)	7% (9)	
Personal history of migraine	213				0.819^¥^
No		58% (19)	57% (17)	62% (93)	
Yes		42% (14)	43% (13)	38% (57)	
Family history of migraine	180				0.019^¥^*
No		48% (12)	36% (10)	63% (80)	
Yes		52% (13)	64% (18)	37% (47)	
Emergency room/urgent care visits/year	192	2	4.5	4	0.411^┼^
Hospital Admissions/year	140	1	1	2	0.729^┼^
Surgery related to CVS	182				0.715^¥^
No		78% (21)	68% (19)	72% (91)	
Yes		22% (6)	32% (9)	28% (36)	
Disability	180				0.421^¥^
No		89% (24)	89% (25)	80%	
Yes		11% (3)	11% (3)	(100) 20% (25)	
Prophylactic medications	177				0.255^¥^
No		4% (1)	12% (3)	4% (5)	
Yes		96% (26)	88% (23)	96% (119)	
Response to therapy	105				0.613^^^
None		7% (1)	24% (4)	20% (15)	
Partial		29% (4)	24% (4)	26% (19)	
Complete		64% (9)	52% (9)	54% (40)	

Median values represented for continuous variables. Numbers after percent’s are frequencies. Tests used: ^┼^Kruskal-Wallis test; ^¥^Fisher’s exact test; ^Proportional odds likelihood ratio test.

*Indicates a P value <0.05.

**Indicates a P value < 0.001.

### Control subjects

A total of 102 control subjects were recruited from UNC for comparison of quantitative pedigree analysis. The median age of controls was 38 (26–53) with 84% (119) females (Table 2). They were significantly older (p = 0.01) and more likely to female (<0.001) than CVS patients. Previously published historical controls (n = 231) were used for comparing the prevalence of genotypes in CVS subgroups. These controls consisted of normal subjects from different geographical locations (North America, Europe, Finland and Italy) but were all Haplogroup H. To reduce potential bias, samples ascertained due to any illness or symptoms, those from self-selected groups (commercial heritage testing), and from small remote islands and/or geographically isolated populations (Iceland and Sardinia) were excluded [7]. Data on age and sex were not collected in this control population.

**Table 2:** **Baseline characteristics and inheritance ratios for CVS patients versus controls**

Variables	CVS (N = 216)	Controls (N = 142)	P-value
Age in years (median, range)	34 (25-46)	38.5 (26-53)	0.011^1*^
Gender			<0.001^2**^
Male	32% (69)	16% (23)	
Female	68% (147)	84% (119)	
Maternal Ratio	1	0.5	<0.001^1**^
Paternal Ratio	0.43	0.29	<0.001^1**^
Matrilineal Inheritance Ratio (MIR)	2.2	1.52	0.007^1*^

Median values represented for continuous variables.

Numbers after percents are frequencies.

Tests used: ^1^Wilcoxon test; ^2^Fisher's exact test.

*Indicates a P value <0.05.

**Indicates a P value < 0.001.

### Quantitative Pedigree Analysis and maternal inheritance patterns of functional disorders in patients with CVS and controls

**Figure 1:**
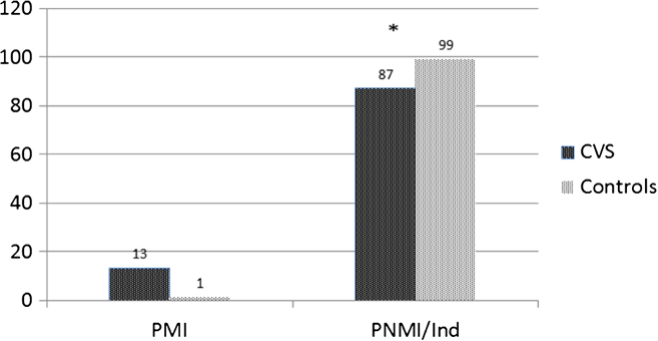
**Inheritance patterns of functional disorders in adults with CVS and controls.** *P value < 0.001 by Proportional odds likelihood ratio test between CVS patients and controls.

The number of functional, neurological and endocrine conditions in both first- and second-degree relatives was determined and the maternal ratio, paternal ratio, and maternal inheritance ratio were calculated using established criteria. The pattern of inheritance (PMI, IND or PNMI) in each individual was determined. Pedigree analysis was also performed in a control population consisting of 102 healthy adults. Table 2 demonstrates the inheritance patterns in adults with CVS versus controls. Adults with CVS were more likely to have a maternal inheritance pattern of functional disorders in comparison to controls (13% vs 1%, p < 0.001, Figure 1). CVS patients also had a higher maternal ratio (1.0 vs 0.5, p <0.001) and a higher MIR (2.2 vs 1.5, p = 0.009). Though controls were older (median age 38.5 vs 33) and more likely to be female (84% vs 69%) when compared to CVS patients, the differences in maternal ratio and MIR were not affected when adjusting for age and gender via regression (Table 2).

Sub-group analysis of inheritance patterns based on onset of disease showed that pediatric-onset CVS patients were more likely to have a maternal inheritance pattern of functional symptomatology compared to adult-onset CVS patients. (25% vs 10%, p = 0.04) The inheritance patterns were also examined by genotype, AT versus non-AT .The maternal ratio, paternal ratio and maternal inheritance ratios were similar in both AT and non-AT subgroups (Table 3). The median MIR in the AT subgroup was higher at 2.58 versus 2.0 in the non-AT group but did not reach statistical significance. Nineteen % (3) of the AT subgroup had a probable maternal inheritance (PMI) pattern of functional symptomatology compared to 13% (21) in the non-AT group, but this also did not reach statistical significance.

**Table 3:** **Inheritance patterns for AT vs non-AT genotype**

Quantitative pedigree analysis	Non-AT (N = 198)	AT (N =18)	P-value
Maternal Ratio	1	0.835	0.923^1^
Paternal Ratio	0.43	0.39	0.565^1^
Matrilineal Inheritance Ratio	2.04	2.58	0.433^1^
Inheritance pattern			0.984^2^
PNMI	80% (143)	81% (13)	
Ind	8% (15)	0% (0)	
PMI	12% (21)	19% (3)	

Median values represented for continuous variables.

Numbers after percents are frequencies.

Tests used: ^1^Wilcoxon test; ^2^Proportional odds likelihood ratio test.

The inheritance patterns were then examined based on genotype (GT, AT, AC and GC) and no correlation was detected between the patterns of inheritance as determined by QPA for each genotype (Table 4).

**Table 4:** **Inheritance patterns by genotype**

Quantitative pedigree analysis	GC (N = 100)	AC (N = 35)	AT (N =18)	GT (N = 63)	P-value
Maternal Ratio	0.885	1.06	0.835	1	0.303^1^
Paternal Ratio	0.5	0.345	0.39	0.38	0.757^1^
Matrilineal Inheritance Ratio	1.81	2.33	2.58	2.75	0.218^1^
Inheritance pattern					0.217^2^
PNMI	85% (79)	70% (24)	81% (13)	75% (40)	
Ind	7% (6)	15% (5)	0% (0)	8% (4)	
PMI	8% (7)	15% (5)	19% (3)	17% (9)	

Median values represented for continuous variables.

Numbers after percents are frequencies.

Tests used: ^1^Wilcoxon test; ^2^Proportional odds likelihood ratio test.

### Mitochondrial DNA polymorphisms in CVS and controls

Of 216 adults who were genotyped, 16519 T alone was present in 38% (81), 3010A in 25% (53) and both 16519 T and 3010A in 8% (18). Of these, 33% (71) were Haplogroup H, identified by the presence of the 7028C polymorphism Of these 71 patients, 34% (24) had 16519 T, 31% (22) had 3010A and the AT genotype was present in one patient. The prevalence of the various genotypes in haplogroup H CVS subgroups and historical controls was similar and is shown in Table 5. Patients with adolescent-onset CVS were excluded from the analysis as they likely represent an indeterminate group.

**Table 5:** **Odds ratios with 95% CI comparing the prevalence of a genotype between haplotype H CVS subgroups and historical controls**

Outcome	Comparison	Estimate	Confidence interval (CI)	P-value
16519 T vs. C	Pediatric vs. Control	2.33	(0.54 – 10.07)	0.74
16519 T vs. C	Adult vs. Control	1.16	(0.48 – 2.82)	1
16519 T vs. C	Adult vs. Pediatric	0.50	(0.10 – 2.50)	1
3010 A vs. G	Pediatric vs. Control	0.53	(0.09 – 3.18)	1
3010 A vs. G	Adult vs. Control	1.09	(0.46 – 2.56)	1
3010 A vs. G	Adult vs. Pediatric	2.05	(0.30 – 13.85)	1
AT vs. non-AT	Pediatric vs. Control	0.00	(0.00 – Inf)	1
AT vs. non-AT	Adult vs. Control	4.18	(0.10 – 177.81)	1
AT vs. non-AT	Adult vs. Pediatric	Inf	(0.00 – Inf)	1

The type I error rate was controlled at 5% using a single-step multiple testing adjustment based on the multivariate normal distribution in the context of a logistic regression model.

Further subgroup analysis showed that there were no differences in the frequency of 16519 T and 3010A between pediatric and adult-onset CVS patients. The frequency of the AT genotype (16519 T and 3010A SNP’s in the same individual) was also similar in both groups of patients (Table 5). The frequency of the AT genotype in non H patients in our cohort was 12% (n = 17) but we are unable to comment on this as we did not have a non H control group for comparison.

## Discussion

This is the first and largest study to our knowledge in adults with CVS to determine the presence and degree of maternal inheritance in adults with CVS in a systematic manner. A detailed family history was ascertained in both first and second degree relatives of the index patient. We also sought to determine if there were any correlation between inheritance patterns and mitochondrial DNA SNP’s 16519 T and 3010A, which have been associated with increased odds of having CVS in children. The findings of our study reveal that adults with CVS patients have a higher proportion of probable maternal inheritance of various functional disorders in comparison to controls (13% vs 1%, p value <0.0001). There was no association between maternal inheritance patterns and genotype as expected. Restricting our analysis to haplogroup H (71/216, 33%) revealed no significant differences in the prevalence of 16519 T, 3010A and the AT genotype in comparison to historical controls. There was also no difference in the prevalence of these mtDNA SNP’s in adult and pediatric-onset patients. While this was not the primary intent of the study, other important findings in this study include the high percentage of patients (28%) who underwent surgery for CVS symptoms. This underscores the need for prompt diagnosis and appropriate therapy in this patient population.

Quantitative Pedigree Analysis (QPA) is a novel tool used for identifying matrilineal inheritance patterns and we obtained a detailed history of all first and second-degree relatives of adults with CVS in our cohort using a standard questionnaire. This revealed that the vast majority of adults (87%) had a non-maternal inheritance pattern of functional symptomatology. A small subset of adults (13%) had a probable maternal inheritance which was significantly different from controls (13 vs 1%, p < 0.001). However there was no correlation between the patterns of inheritance and mtDNA SNP’s 16519 T and 3010A as expected; this could be explained by the presence of yet unidentified mitochondrial polymorphisms beyond the two that were studied. While DNA methylation may be another possible explanation this would be less likely and needs to be explored in future studies.

Our genetic analysis of Haplogroup H adults with CVS (33%, 71) did not show any differences in the frequency of 16519 T, 3010A and the AT genotype between our cohort and historical controls. We were unable to comment on our non-H group as we did not have a control non-H population readily available for comparison. Age of onset was also not a determinant of the presence of the mtDNA SNP’s as reported in a previous study. The study by McCallum et al. revealed that pediatric onset CVS patients were more likely to have 16519 T and the AT genotype. One possible explanation for these differences between our study and the previous one may be due to the fact that most of the childhood onset patients in the above mentioned study were recruited through a genetic clinic which may have resulted in a referral bias. Twelve % (17) of patients in the non H group had the AT genotype but the significance of these results is unclear with this present study. Most of the studies in CVS have been performed in Haplogroup H patients as they are genetically homogenous.

A recent study by Camilleri et al. examined the frequency of irritable bowel syndrome (IBS) in both Haplogroup H and non- H patients. This interesting study revealed that the IBS –C and IBS with alternating constipation and diarrhea was more prevalent in non H individuals. In this same study, Haplogroup H individuals had higher maximal tolerated volumes (lower satiety) and 3010A in these individuals was associated with abdominal pain/dyspepsia and more rapid emptying [13]. The frequency of the AT genotype in this non H group was not readily available but future collaborative studies pooling such data would serve to make more meaningful comparisons.

The strengths of our study are that this represents a large sample of adults with CVS with extensive clinical information obtained prospectively. Pedigree analysis was conducted using a standard script, ensuring uniformity in data collection. Limitations include the possibility of recall bias when obtaining family histories and that most of the information was usually gleaned from the patients and their mothers, which may have led to missing information on the paternal side. However, every effort was made to address this and patients were called on multiple occasions if necessary to clarify the family history. However, the same limitations apply to the control group as well and are unlikely to have influenced overall results. Other limitations include the lack of a gold standard for diagnosing CVS as with other functional disorders. While this was a large heterogeneous group of CVS patients, we cannot exclude a referral bias with sicker patients being included. Arguably these are the very patients who would need more aggressive treatment, and understanding the mechanisms underlying CVS would be even more important with this population. Other considerations are that we chose to include only haplogroup H patients for genotype comparison with controls as non-H control data was not readily available. Given the changing demographics of the US population, future studies should be extended to include both groups of patients. The presence of a high degree of matrilineal inheritance of functional disorders was not associated with a specific genotype as expected and may be due to yet unidentified mtDNA sequences. Other factors beyond the mtDNA sequence, possibly including nuclear-encoded mitochondrial genes, other genes, and the environment may play a larger role in the pathophysiology of CVS in adults as opposed to children.

## Conclusions

Our study demonstrates that CVS in a subset of adults is associated with a maternal inheritance pattern of functional GI disorders as seen in children but is not associated with 16519 T, 3010 A or the AT genotype. A matrilineal inheritance pattern was higher in pediatric onset CVS compared to adult onset CVS. However, onset of disease did not have any correlation with the frequency of the mtDNA SNP’s. While this study offers some insight into the genetic background of CVS, further studies examining the entire mtDNA, as well as the full mitochondrial exome (the full mtDNA sequence plus nuclear genes encoding mitochondrial proteins) are warranted.

## Electronic supplementary material

Additional file 1: **Script for pedigree analysis.** (PDF 37 KB)

## Abbreviations

CVS: Cyclic vomiting syndrome
mtDNA SNP’s: Mitochondrial DNA single-nucleotide polymorphisms
QPA: Quantitative pedigree analysis
RFLP: Restriction fragment length polymorphism
KSP: Karolinska Scales of Personality
CVSA: Cyclic vomiting syndrome association
PMI: Probable maternal inheritance
IR: Maternal inheritance ratio
Ind: Indeterminate
PNMI: Probable non-maternal inheritance
UNC: University of North Carolina
IRB: Institutional Review Board.
