# Supplementary material for: Quantitative pedigree analysis and mitochondrial DNA sequence variants in adults with cyclic vomiting syndrome
Source: BMC Gastroenterol. 2014 Oct 21;14:181. doi: 10.1186/1471-230X-14-181 (PMC4287476; doi:10.1186/1471-230X-14-181)
Supplement: Supplementary file 1 — Additional file 1: Script for pedigree analysis. (PDF 37 KB) [file 12876_2014_1208_MOESM1_ESM.pdf]

Scripted Pedigree for Quantitative Maternal Inheritance Ratio Analysis

All positive answers are followed up with additional questions, especially in terms of age of onset, duration, severity, diagnoses given, etc.

General

1. Have you ever been hospitalized (except for giving birth)?
2. Have you had any other chronic illnesses or medical conditions in your life, especially ones that required hospitalization or medical intervention?
3. Have you been diagnosed with or experienced chronic fatigue that inhibited your ability to perform everyday tasks, such as grocery shopping?
4. Were you born with any birth defects?

Dysautonomia

1. Have you experienced chronic headaches or migraines?
2. Have you ever had episodes of vomiting, an episode of severe vomiting that required IV treatment, or have you been given a diagnosis of cyclic vomiting or hyperemesis gravidum?
3. Do you have or have you had in the past chronic pain, such as pain in the chest, abdomen, arms, legs or joints?
4. Do you frequently experience numbness or tingling anywhere in your body?
5. Have you been diagnosed with fibromyalgia or reflex sympathetic dystrophy?
6. Have you had changes in skin color (including rash), size (swelling), or temperature?
7. Have you experienced any gut or bowel problems such as GERD (reflux, heartburn), delayed gastric emptying, irritable bowel, diarrhea, constipation or bloating?
8. Have you experienced unusually high, low, or fluctuating vital sign changes such as changes in heart rate, breathing rate, blood pressure, or temperature regulation? [*Hypertension excluded*]
9. Do you get out of bed frequently at night to use the restroom?

Cognitive/Psychiatric/Behavioral

1. Do you have any learning disabilities or did you have any learning problems in school?
2. Have you been diagnosed with attention deficit disorder, with or without hyperactivity? Do you have substantial problems concentrating at work or school?
3. Have you been diagnosed with autism, autistic features, or Asperger syndrome?
4. Have you ever had an episode of depression?
5. Have you ever been treated for anxiety or panic disorders?
6. Do you have any other psychiatric or behavioral conditions?

Muscular

1. Have you ever had hypotonia? Have you had muscle weakness, atrophy or cramps?
2. Have you been diagnosed with strabismus (crosseyedness)?
3. Have you been diagnosed with cardiomyopathy (failure of the heart muscle)? [*CAD excluded*]

Neurological

1. Do you have balance problems, ataxia, incoordination or cerebral palsy?
2. Have you ever had a stroke? Have you ever lost any abilities that you had before?
3. Have you ever had a seizure or fainting spell?
4. Do you have any hearing problems?
5. Do you have tinnitus (ringing in the ears)?
6. Have you been diagnosed with a retinal disease (an eye condition such as retinitis pigmentosa or optic neuropathy)? Have you had substantial problems with vision that were not correctable with glasses/contacts?

Endocrine

1. Have you been diagnosed with hypoglycemia (low blood sugar)? Do you get any unusual symptoms when you haven't eaten in a while?
2. Do you have a thyroid problem?
3. Have you been diagnosed with growth retardation or failure to thrive?
4. Have you been diagnosed with any other hormonal conditions? [*Diabetes mellitus types 1 and 2 excluded*]
